# Suburban Doctors and Suburban Hospitals

**Published:** 1891-05-16

**Authors:** 


### The Hospital

A Weekly Journal of

Science, Medicine, Nursing, and Philanthropy.

*For Hospitals, Asylums, Infirmaries, Dispensaries, Medical Practitioners, Students, Nurses, and the Charitable Public.*

Vol. X.—No. 242.] [May 16, 1891.

### Suburban Doctors and Suburban Hospitals.

The suburban medical preserves of prosperous practitioners are being rapidly invaded by the ubiquitous hospital system. It would be almost impossible to get sufficient money to build a new hospital in the heart of London; but there is very little difficulty in raising the necessary funds for the erection and equipment of suburban hospitals in those districts where they are obviously needed. In the northern suburbs of London alone there have been no fewer than three general hospitals erected during the last few years, viz., the Metropolitan in the Kingsland Road, the Great Northern Central in the Holloway Road, and the North-West London in Kentish Town.

It would be affectation to affirm that these new hospitals are regarded with unmixed satisfaction by the medical practitioners in their vicinity. A few of the more popular doctors, it is true, can afford to be indifferent to the near presence of a hospital. They have pleasant manners, competent professional skill, and assured popularity. No competing hospital can touch either their dignity or their pockets. But such practitioners constitute a small minority. The majority of their brethren are by no means able to take up this attitude of prosperous indifference. They have a strong conviction that a hospital in their neighbourhood injures them very seriously, both in their personal dignity and in their financial prosperity. The hospital, by means of its unpaid medical staff, undersells them in point of fees; and it overshadows their professional status by the superior distinction of its honorary medical and surgical officers. It comes to this in many a man's case, that he feels with disgust that he can never be anything but a drudge and a slave. Such are the arrangements of hospitals, both in the centre and in the outlying parts of London, that even a Jenner, an Andrew Clark, or a Paget, practising in the suburbs, would find himself inferior in the general estimation to many a young hospital doctor of thirty, who had neither knowledge, experience, nor capacity to recommend him, but only assurance and a respectable degree.

Suburban doctors are not patient under these circumstances. They would show themselves to be both spiritless and stupid if they were. To a common mind, a moderate income with moderate work, in a respectable suburb, is the maximum of happiness. But there surely must be, even among suburban medical practitioners, here and there a man of philosophic intellect, of strong personality, and of highly trained professional skill and experience. Such a man does himself and his profession an injustice if he submits tamely to the estimate which is formed of him by the commonplace hospital physician, and which is industriously imposed upon the public judgment by all sorts of direct and indirect means. The affairs of medical practice, and of hospitals as standing in some sort at the head of medical practice, ought to be so arranged as that a man of this kind should be called to his proper position in the profession, and should shed the lustre of his greater dignity and worth upon the hospital of his neighbourhood.

Those who are familiar with the internal affairs of some of the London hospitals know well what kind of stuff it is of which a considerable part of their honorary medical staff is composed. It is not merely that some hospitals, which can reckon their physicians and surgeons by the dozen, have not a single man of distinction among them all; but it is the plain truth that a considerable number, at any rate of metropolitan hospitals, are manned by men whose professional capacity reaches a poor general average, and whose intellectual culture is of the most limited and illiberal kind. These men play havoc in the profession; they disgust general practitioners by their vulgar affectation of superiority, and they bring the name of "physician" into public contempt by their lack of that peculiar dignity which culture and character can alone bestow.

Hopelessness is the state of mind which many able suburban practitioners indulge under these circumstances: hopelessness and disgust. They are aware that their professional brethren, who are already in secure possession of hospital appointments, are the very last persons in the world to discover any merit in even the ablest outsider. If such outsider were an Aristotle, a Bacon, a Hunter, a Harvey, a Jenner, and a Christison rolled into one, is there a single medical staff in London which would see in him anything but a "general practitioner?" There is a good deal of dishonour here! But the dishonour belongs to the blind hospital staff, not to the general practitioner. There are some who think that the lay members of the hospital committee, seeing that they live in the neighbourhood of the hospital, ought to have a keen eye for local practitioners of unusual merit; and ought, when they find such, to invite them to fill hospital appointments. That would be a reasonable method of procedure. It is obvious that if a man treats the wealthier and the middling classes with skill and success, he is very likely, indeed, to treat the poorer persons who come to hospitals with equal skill and success. This is so self evident, that it would be foolish to waste time in a single word of argument.

But why then does no hospital committee at least once in a way—say once in a century—put its finger upon a local man of unusual merit, and appoint him physician or surgeon to the local hospital? Why? Because the members of the committee have not sufficient original intelligence to recognise such a man when they see him; and because, even if they had the intelligence, they have not the courage and independence of mind to face the feeble disapproval of the least intelligent members of their medical staff. The medical charities of this country are, in many respects, noble institutions, but "intellectual light" and "sweet reasonableness" have attained to very microscopic proportions in some departments of their management.

One of the most burning of hospital questions at the present moment is this: Whether the best interests of the public and the medical profession are served by making voluntary hospitals close preserves for practitioners who start the world with a little more money than their fellows? Can medicine afford to wear the cast-off clothing of the army, and to maintain a system of indirect purchase? It certainly cannot. Judging from the recent conduct of Mr. Stanhope at the War Office, and from the evident neglect of the medical profession, both by Governments and the higher social leaders, it is evident that medicine is by no means holding its own in the general struggle for public recognition and status. Why is this? It can only be because many of the more prominent members of the profession are deficient in those qualities which place men on an intellectual and practical equality with the best and most capable of their fellow-countrymen. It is not the middle ranks of the profession which are losing ground. Never before did the really worthy family practitioner hold so high a position in the estimation of his social equals as he does at thepresent time. But the same can by no means be said with truth of many of those prominent physicians and surgeons who are permitted to write prescriptions for the great, and to bandage or even cut off their legs and arms, but who in other respects are kept at a level of recognition which is of anything but a flattering kind.

The truth is that medicine can no more afford to keep its best men in the back ground than can an army to depress its best generals, or a State its most capable politicians. The regulations at the hospitals, which have for the most part been made by a mediocrity that is secure in the interests of a mediocrity which is anxious to become secure, are obsolete, stupid, and injurious. The committees of management which permit them to continue ought to be ashamed of them, and the physicians and surgeons who know their true value ought to be ashamed of them still more. Every commissioned officer of Her Majesty's service may properly aspire to the command of Her Majesty's greatest army, and his hope of success lies in attention to duty, practical capacity, and superior intelligence. Every fully qualified medical man is already a commissioned officer in medicine. It is to the interest of the whole profession and of the public to clear the way to the very highest position for him who shows superior attention to duty, superior practical capacity, and superior intelligence. As a matter of fact, ten thousand flaming swords bar the way of every capable man who is not prepared at all risks to pass through one particular gateway—a gateway that is sometimes made so very narrow that no man of robust intellectual and moral proportions can bring himself to the task of squeezing through it.

